# A different perspective on cervical X-ray parameters in nTOS: could it play a role in pathophysiology?

**DOI:** 10.1186/s13018-026-06694-7

**Published:** 2026-02-02

**Authors:** Ugur Bezirgan, Orhun Eray Bozkurt, Ebru Dumlupinar, Mehmet Armangil

**Affiliations:** 1 Faculty of Medicine, Orthopedics and Traumatology Department, Ankara University, Hand Surgery Unit, Ankara, Turkey; 2 Orthopedics and Traumatology Department, Kulu State Hospital, Konya, Turkey; 3 Faculty of Medicine, Biostatistics Department, Ankara University, Ankara, Turkey

**Keywords:** Neurogenic thoracic outlet syndrome, Neck tilt, T1 transverse process angle, Fibrous band, Cervical X-ray

## Abstract

**Introduction:**

Neurogenic Thoracic Outlet Syndrome (nTOS) is characterized by compression of neural structures within the interscalene triangle of the thoracic outlet, with anatomical variations frequently implicated in its etiology. In patients with TOS, spinal nerves pass through a narrow anatomical space as they exit the foramen and traverse the thoracic inlet. The tension of the suprapleural bands extending between the C7 transverse process and the first rib, along with changes in neck position and certain anatomical variations, can lead to compression of the lower trunk. The condition of this anatomical space, where compression occurs, is influenced by the relationship between the lower cervical vertebrae and the first rib. This study aims to determine whether these cervicothoracic angles could lead to lower trunk compression as it passes over the suprapleural membrane. It is hypothesized that this radiological variation may present a higher likelihood of causing compression in TOS patients compared to the normal population.

**Methods:**

This retrospective study included patients diagnosed with neurogenic TOS who underwent surgery between 2015 and 2023, patients managed with physical therapy without surgery, and a matched control group. The control group consisted of patients presenting to the orthopedic clinic with neck pain but without any pathological findings. Cervical anteroposterior and lateral radiographs were evaluated to measure T1 slope, cervical inclination, the length of the T1 transverse process, and the angle between the T1 transverse process and the T1 vertebral body. We hypothesize that differences in these radiological parameters, often assessed in patients with cervical spondylosis, might contribute to nerve compression at the thoracic outlet. These parameters were compared between groups. Thoracic Outlet Syndrome Index (TOSI) scores and surgical approaches were documented for the operated patients.

**Results:**

The study included 52 patients with a mean age of 34.23 ± 11.15 years. Of these, 24 patients were in the TOS group, and 28 were in the control group. Sixteen of the 24 TOS patients underwent surgery, while 8 were managed with physical therapy. Significant differences were observed between the TOS and control groups in cervical inclination and T1 transverse process angle (*p* = 0.04, *p* = 0.004). However, no significant relationship was found between T1 slope, T1 transverse process length, and TOS. Cervical inclination was 43 degrees in the TOS group and 48 degrees in the control group. The T1 transverse process angle was 106.5 degrees in the TOS group and 116.5 degrees in the control group. Among the 16 operated patients, 13 (81.25%) underwent a supraclavicular approach, 2 (12.5%) had a combined supraclavicular and pectoralis minor approach, and 1 (6.25%) underwent a pectoralis minor approach. Supraclavicular surgeries included fibrous band excision and anterior and middle scalenectomy. The preoperative and postoperative TOSI scores for the 16 operated patients were 26.2 and 2.23, respectively, with a statistically significant difference (*p* < 0.001).

**Discussions:**

Increased tension in the fibrous bands over the Sibson-Truffert fascia, exacerbated by poor neck posture, may contribute to lower trunk compression in TOS. The literature supports treatment through excision of these bands via a supraclavicular approach, yielding clinically satisfactory outcomes.

**Conclusion:**

In conclusion, demonstrating cervical inclination and T1 transverse process angle preoperatively as indicators of lower trunk compression may predict favorable outcomes with supraclavicular surgery.

**Supplementary Information:**

The online version contains supplementary material available at 10.1186/s13018-026-06694-7.

## Introduction

Neurogenic Thoracic Outlet Syndrome (nTOS) presents as a complex clinical condition caused by the compression of neurovascular structures in the thoracic outlet region. The diagnosis of nTOS relies on clinical suspicion, pattern recognition, and the exclusion of more common conditions with overlapping features. In most patients, the diagnosis of neurogenic TOS can be established or ruled out through clinical history, symptom characterization, and physical examination [1]. It primarily manifests with symptoms such as numbness, tightness, and pain in the shoulder, arm, and hand. Notably, cases involving numbness in all fingers can be observed not only in distal entrapment neuropathies but also in nTOS. The TOS cadaver model demonstrated that the etiology of compressive neuropathies, such as cubital tunnel syndrome or carpal tunnel syndrome, is not solely linked to the compressive lesion on the nerve but is also associated with fibrosis and traction neuropathy [2]. These symptoms become more pronounced in the mornings and significantly impact the patients’ quality of daily life.

A significant portion of TOS patients are initially evaluated with suspicion of cervical disc pathologies and undergo prolonged conservative treatment accordingly. However, the failure of these treatments and the persistence of symptoms often suggest the presence of an underlying pathology. Most patients are initially diagnosed with cervical spondylosis, and resistance to physical therapy leads to reconsideration of the diagnosis, ultimately pointing to nTOS [3].

The etiology of TOS is multifactorial, with factors such as abnormal fibrotic bands, tight or hypertrophic muscles, trauma sequelae, and poor posture playing a prominent role [4]. It is well known that the brachial plexus is susceptible to compression as it passes through the narrow anatomical space between the shoulder girdle and the upper thorax, influenced by surrounding bony and fibromuscular structures. Postural abnormalities, particularly conditions such as ‘upper crossed syndrome,’ can increase compression on the brachial plexus nerves, thereby contributing to the development of this syndrome [5, 6]. Mechanisms such as scapular band ptosis, narrowing of the costoclavicular space, and scalene muscle shortening can affect not only the nerves but also the blood vessels.

It has previously been established that there is a strong correlation between cervical sagittal parameters and Neck Disability Index (NDI) scores [7]. This study investigates the effect of anatomical and radiological parameters in the cervical spine and thoracic outlet region on lower trunk compression in patients with nTOS. The findings are expected to provide guidance in the diagnosis and surgical treatment process of nTOS.

## Methods

**Fig. 1:**
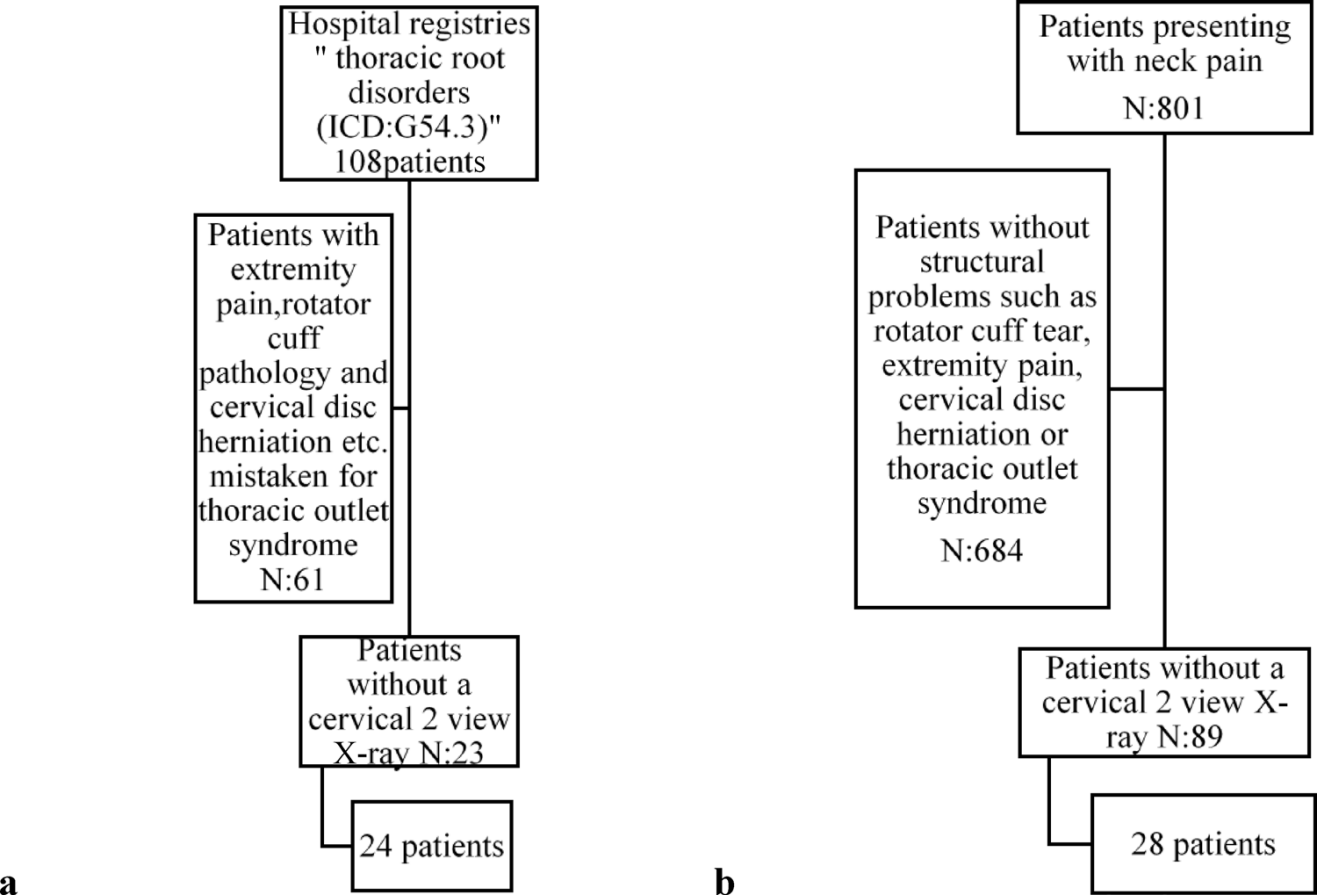
Flow chart of the patients included in the study. **a** Flow diagram of the nTOS group included in the study. **b** Flowdiagram of the control group included in the study

This study retrospectively evaluated 24 patients who were diagnosed with nTOS and underwent surgery or were managed with physical therapy without surgery between 2015 and 2023. A control group of 28 patients who presented to the Orthopedics clinic with neck pain but no pathology was found was also included (Fig. 1). The study was approved by the institutional ethics committee (I03-255-24). Electronic medical records of all patients were reviewed, and radiological images were analyzed. The surgical approaches of the patients who underwent surgery, along with their preoperative and postoperative TOSI scores, were recorded [8]. Patients in the control group were selected after excluding cervical disc pathologies and other structural issues. Cervical anteroposterior and lateral radiographs of all patients included in the study were thoroughly examined.

**Fig. 2:**
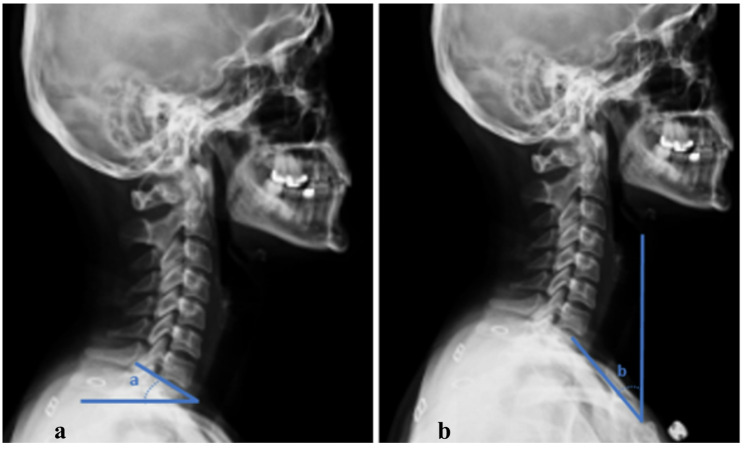
Measurement of T1 slope (**a**) and neck tilt (**b**) on a lateral cervical X-ray. T1 slope is defined as the angle between the superior endplate of the T1 vertebra and the horizontal plane on a standing lateral radiograph. T1 tilt is defined as the angle between a line connecting the midpoint of the superior endplate of T1 to the upper end of the sternum (manubrium) and the vertical axis

**Fig. 3:**
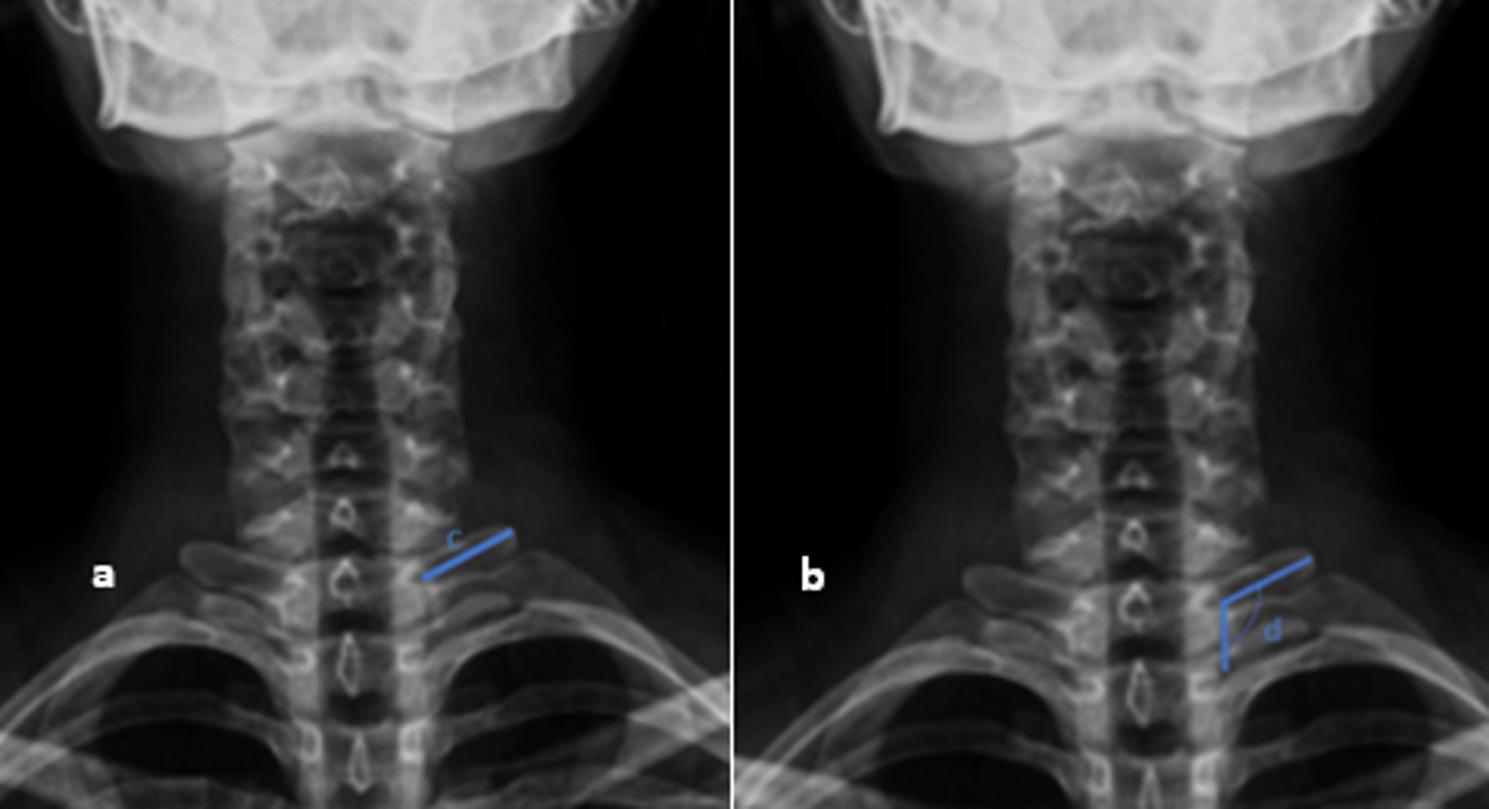
Measurement of T1 transverse process length and angle with vertebral body on cervical AP X-ray (**a**) The length of the T1 transverse process was defined as the distance between the lateral margin of the T1 vertebral body and the most lateral tip of the T1 transverse process. (**b**) The angle between the T1 transverse process and the T1 vertebral body was measured as the angle formed between the longitudinal axis of the T1 vertebral body and its lateral margin

On the radiographs, T1 slope, neck tilt, length of the T1 transverse process, and the angle between the T1 transverse process and the T1 body were measured [9, 10] (Figs. 2 and 3). Each parameter was measured twice, and the averages were calculated. The measurements were performed by an independent observer. The differences between these parameters in the TOS and control groups were evaluated.

The surgical approaches used in the operated patients were analyzed in detail. Supraclavicular approaches were predominantly employed, although alternative surgical techniques were utilized in certain cases. The preoperative and postoperative TOSI scores of these patients were compared to assess the effectiveness of the surgical intervention. Radiological and clinical data were interpreted to examine the relationship between anatomical and radiological markers and lower trunk compression in nTOS patients.

### Statistical analysis

Descriptive statistics were presented as mean ± standard deviation for the variables distributed normally and as median (min, max, IQR) for the variables distributed not normally, whereas they were presented as number and percentage (%) for nominal variables. The significance of the difference between the groups in terms of the median values was analyzed by Mann-Whitney U Test and Wilcoxon Signed Rank Test. Categorical variables were evaluated using Pearson’s Chi-Square Test and Fisher’s Exact Test. A p value of less than 0.05 was considered statistically significant and the analyses were conducted using the Statistical Package for Social Sciences (SPSS, Version 11.5, Chicago, IL).

## Results

A total of 52 patients were included in the study; 24 of them were in the nTOS group, while 28 were in the control group. The mean age of patients diagnosed with nTOS was calculated as 34.23 ± 11.15 years.

Of the 24 patients in the nTOS group, 16 underwent surgical treatment, while 8 were followed up with physical therapy (Tables 1 and 2). Radiological measurements revealed statistically significant differences between the nTOS and control groups in terms of neck tilt and T1 transverse process angle (respectively *p* = 0,04 ve *p* = 0,004) (Table 3). Neck tilt was measured as 43 degrees in the nTOS group and 48 degrees in the control group. The T1 transverse process angle was calculated as an average of 106.5 degrees in the nTOS group and 116.5 degrees in the control group (Table 3) .

No significant difference was found between the two groups in terms of T1 tilt and the length of the T1 transverse process. Among the 16 surgically treated patients, 13 (81.25%) underwent a supraclavicular approach, 2 (12.5%) underwent a combination of supraclavicular and pectoralis minor approaches, and 1 (6.25%) underwent only a pectoralis minor approach. In supraclavicular surgeries, fibrous band excision was performed along with anterior and middle scalenectomy. The TOSI scores of surgically treated patients decreased from a preoperative average of 26.2 to 2.23 postoperatively, and this difference was found to be statistically significant. (*p* < 0,001) (Table 4).

## Discussion

**Fig. 4:**
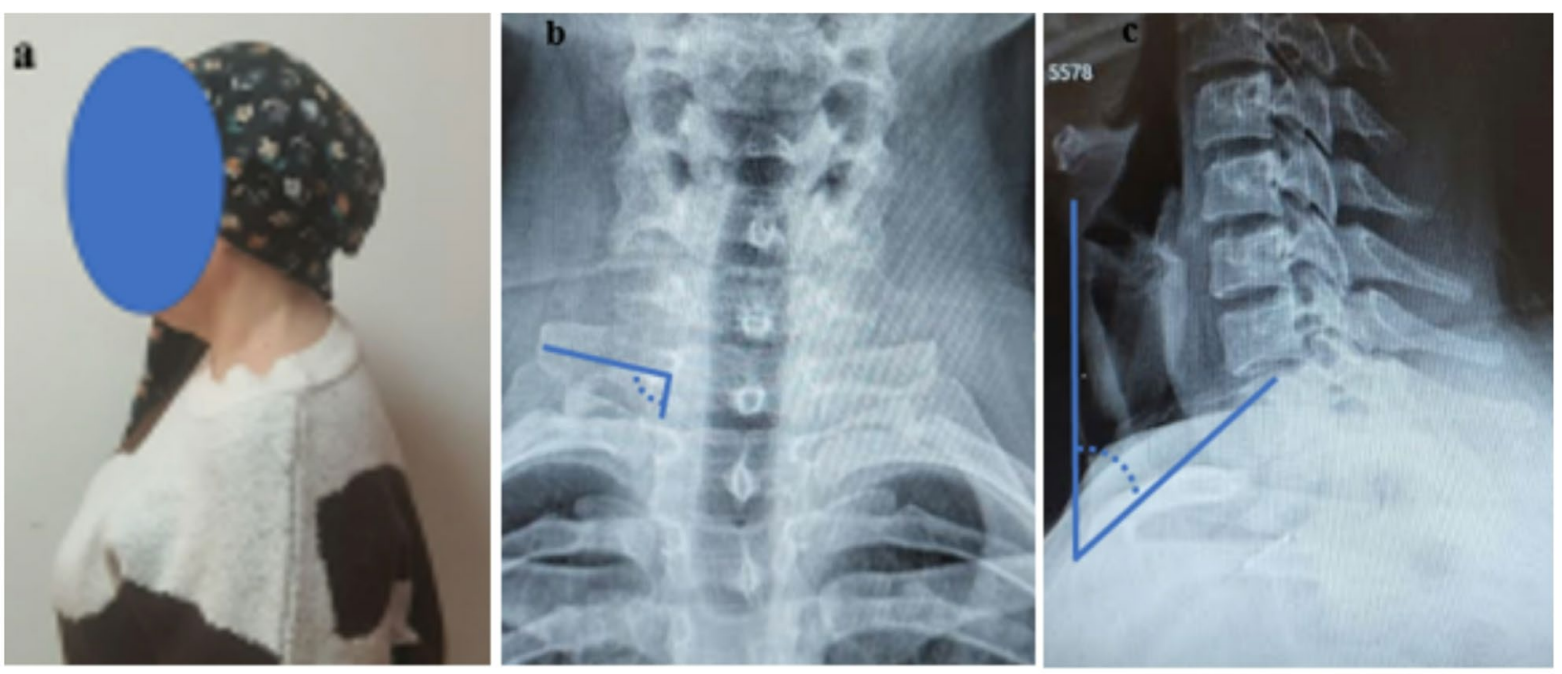
The sagittal plane flexion posture image of a patient (**a**) followed up with nTOS in our clinic. T1 transverse process angle was measured as 100° (**b**) and The neck tilt was measured as 44° (**c**)

This study demonstrated that neck tilt and T1 transverse process angle are significant radiological parameters that may contribute to lower trunk compression in patients with nTOS. In the nTOS group, the lower neck tilt compared to the control group and the narrower T1 transverse process angle suggest that structural compression in the thoracic outlet region may be associated not only with anatomical variations but also with postural changes in the cervical region (Fig. 4). This finding provides a new perspective on the pathophysiology of nTOS and highlights that cervical X-ray analysis could be an important tool in the evaluation of this syndrome.

In particular, our study goes beyond the existing approaches in the literature regarding the radiological evaluation of nTOS [11]. For example, there is currently no definitive imaging test to diagnose TOS resulting from the narrowing of the costoclavicular space [12]. This study has demonstrated that cervical X-ray analysis is an effective method for revealing dynamic and postural causes beyond anatomical variations. Previously, the pathophysiology of nTOS was typically discussed through anatomical structures such as cervical ribs, abnormal first ribs, and fibrous bands. However, this study clearly demonstrates that poor posture and imbalances in the head-shoulder axis contribute to narrowing of the thoracic outlet, thereby leading to lower trunk compression [13]. The absence of other studies in the literature that address the impact of cervical X-ray analysis on nTOS in this context positions this study as pioneering in the field. In this regard, we believe that our study makes a significant contribution to the scientific community.

The results obtained in our study suggest that the combination of tension in the fibrous bands on the Sibson-Truffert fascia and poor neck posture may contribute to lower trunk compression in nTOS [14]. While the literature reports that surgeries performed with a supraclavicular approach provide successful treatment options for such compressions, the findings in our study support the effectiveness of these surgical approaches [15, 16]. In particular, procedures such as fibrous band excision and anterior-middle scalenectomy performed in supraclavicular surgeries have significantly improved symptoms by reducing pressure on the brachial plexus. Careful evaluation of preoperative radiological parameters plays a critical role in determining the appropriate surgical treatment option and achieving successful outcomes.

During surgery, the division of the scalene muscles and, when necessary, the pectoralis minor tendon addresses the underlying cause of narrowing in the thoracic outlet by eliminating involuntary muscle contractions. This approach has contributed to significant symptomatic improvement in patients and the achievement of favorable TOSI scores.

An elongated C7 transverse process and T1 vertebral slope are not considered significant in the evaluation of patients with nTOS. This is because these anatomical parameters cannot be corrected through conservative or surgical treatment; the former can only be altered by surgical excision, while the latter is generally expected to remain unchanged with treatment. However, the primary focus of this study is neck tilt, which is expected to improve with treatment. Neck tilt plays a critical role in evaluating the response to treatment and emerges as a key parameter in determining the symptomatic improvement of patients.

The impact of poor neck posture on nTOS remains unclear. There is some ambiguity in the literature regarding whether this postural dysfunction is a consequence or a cause of nTOS [17, 18]. Changes in neck position are thought to exacerbate structural compression in the thoracic outlet region, leading to lower trunk compression. However, further data are needed to understand how these postural dysfunctions influence symptoms over time and which factors play a more dominant role. Therefore, larger prospective studies are necessary to reach a definitive conclusion. Such studies will provide a more detailed understanding of the pathophysiology and help shape treatment strategies.

In the literature, there are studies indicating that chiropractic spinal manipulation corrects cervical extension deficiencies and supports the reformation of cervical lordosis [19, 20]. However, the lack of data from follow-up radiographs to demonstrate this anatomical improvement in our treated patients who showed significant symptomatic relief is a major limitation of our study. This issue should be considered both to evaluate treatment efficacy more objectively and to enhance the comparability of the results with the existing literature. In future studies, the inclusion of radiological evaluations will be beneficial in addressing this limitation.

## Conclusion

In conclusion, cervical parameters such as neck tilt and T1 transverse process angle are considered important indicators for identifying the mechanisms leading to lower trunk compression. These findings emphasize the necessity of considering these parameters in clinical evaluation and the surgical treatment process. An important contribution of our study is that cervical X-ray analysis reveals that, beyond anatomical variations, postural and dynamic factors also play a significant role in nTOS patients. As the first study in this area, our work enhances its scientific value and leads to a new understanding in the diagnosis and treatment of nTOS.

**Table 1:** Demographic and clinical characteristics of patients

Age, mean ± SD	34.23 ± 11.15
*Sex, n (%)*	
Female	43 (82.7)
Male	9 (17.3)
*Side, n(%)*	
Right	33 (63.5)
Left	19 (36.5)
*TOS, n (%)*	
Absent	28 (53.8)
Present	24 (46.2)

SD, standart deviation; TOS, thoracic outlet syndrome

**Table 2:** The results of the patients’ radiological and TOSI parameters

Neck Tilt, mean ± SD	45.57 ± 8.48
T1 slope, mean ± SD	23.9 ± 8.32
T1 transverse process angle, mean ± SD	112.73 ± 11.29
T1 transverse process length, mean ± SD	2.78 ± 0.37
Preoperative TOSI score, median (min, max, IQR)	26.2 (21.6, 29.8, 4.8)
Postoperative TOSI score, median (min, max, IQR)	2.23 (0, 8.13, 5.41)

min, minimum; max, maximum; IQR, interquartile range, SD, standart deviation; TOSI, thoracic outlet syndrome index

**Table 3:** Comparison of demographic and radiological data between the TOS and control groups

	TOS		
	Absent (*n* = 28)	Present (*n* = 24)	*p* value
*Sex, n(%)*			
Female	23 (82.1)	20 (83.3)	0.999^a^
Male	5 (17.9)	4 (16.7)	
*Side, n(%)*			
Right	21 (75)	12 (50)	0.062^b^
Left	7 (25)	12 (50)	
Neck tilt, median (min, max, IQR)	48 (31, 72, 8.75)	43 (32, 62, 10.25)	0.04^c^
T1 slope, median (min, max, IQR)	22.5 (8, 46, 11)	26.5 (11, 38, 15.25)	0.496^c^
T1 transverse process angle, median (min, max, IQR)	116.5 (101, 146, 15)	106.5 (93, 131, 11.5)	0.004^c^
T1 transverse process length, median (min, max, IQR)	2.64 (1.74, 3.36, 0.68)	2.85 (2.12, 3.51, 0.59)	0.075^c^

min, minimum; max, maximum; TOS, thoracic outlet syndrome; IQR, interquartile range

^a^Fisher’s exact test

^b^Pearson chi-square test

^c^Mann-whitney U test

**Table 4:** Comparison of TOSI score between the TOS and control groups

	TOS		
	Preoperative (*n* = 16)	Postoperative (*n* = 16)	*p* value
TOSI score, median (min, max, IQR)	26.2 (21.6, 29.8, 4.8)	2.23 (0, 8.13, 5.41)	< 0.001^d^

min, minimum; max, maximum; TOS, thoracic outlet syndrome; TOSI, thoracic outlet syndrome index; IQR, interquartile range

^d^Wilcoxon signed rank test

## Supplementary Information

Below is the link to the electronic supplementary material.

Supplementary Material 1

## Data Availability

No datasets were generated or analysed during the current study.

## References

[1] Thompson RW. Diagnosis of Neurogenic Thoracic outlet syndrome: 2016 Consensus Guidelines and Other Strategies. In: Illig KA, et al. editors. Thoracic outlet syndrome. Cham: Springer; 2021. pp. 67–97. 10.1007/978-3-030-55073-8_9.

[2] Tremblais L, Rutka V, Cievet-Bonfils M, et al. The consequences of a thoracic outlet syndrome’s entrapment model on the biomechanics of the ulnar nerve—Cadaveric study. J Hand Ther. 2023;36(3):658–64. 36289037 10.1016/j.jht.2022.09.007

[3] Tek ŞT, Fırat T, Cangır AK. Investigation of cervical joint position sense in patients with thoracic outlet syndrome. J Hacettepe Univ Phys Therapy Rehabilitation Fac. 2024;3(2):17–23.

[4] Sanders RJ, Donahue DM. Pathology and Pathophysiology of NTOS, in Thoracic Outlet Syndrome. 2021. pp. 53–60.

[5] Bellin D,TAM, Cheung DMY. Spinal manipulative therapy for atypical cervicogenic symptoms: A review. J Orthop Sports Med. 2023;5:56–61.

[6] Asad A, Farooq N, Kafeel S, et al. Association of upper crossed syndrome and posture among general population having neck pain in Islamabad. J Rehman Med Inst. 2021;7(2):07–11.

[7] Xu Y, Liu S, Wang F, et al. Cervical sagittal parameters were closely related to neck disability index score after anterior cervical decompression and fusion. J Orthop Surg Res. 2020;15:1–12. 32795309 10.1186/s13018-020-01836-x PMC7427731

[8] Ruopsa N, Vastamäki H, Ristolainen L, et al. Convergent validity of thoracic outlet syndrome index (TOSI). Phys Activity Health. 2022;6(1):16–25.

[9] Zhang J, Zhang C, Zhong W et al. Validity and reliability of a novel iPhone method to rapidly measure cervical sagittal parameters. Sci Rep. 2022;12(1):19579. 10.1038/s41598-022-21660-z PMC9666521 36380107

[10] Yu C, Chong XZ, Liu S, Liao, et al. Risk factors for recurrent L5–S1 disc herniationafter percutaneous endoscopic transforaminaldiscectomy: A retrospective study. Med Sci Monitor. 2020;26:e919888-1. 10.12659/MSM.919888 PMC7133417 32210223

[11] Khalilzadeh O, Glover M, Torriani M, et al. Imaging assessment of thoracic outlet syndrome. Torac Surg Clin. 2021;31(1):19–25. 10.1016/j.thorsurg.2020.09.002 33220768

[12] Duarte FH, Zerati AE, Gornati, et al. Normal costoclavicular distance as a standard in the radiological evaluation of thoracic outlet syndrome in the costoclavicular space. Ann Vasc Surg. 2021;72:138–46. 33160055 10.1016/j.avsg.2020.09.060

[13] Teijink SBJ, Pesser N, Goeteyn J et al. General Overview and Diagnostic (Imaging) Techniques for Neurogenic Thoracic Outlet Syndrome. Diagnostics (Basel). 2023;4(13):1625. 10.3390/diagnostics13091625 PMC10178617 37175016

[14] Boretto JG, Rellán I, De Cicco FL. Compression neuropathy: thoracic outlet Syndrome, in clinical examination of the hand. CRC; 2022. pp. 216–23.

[15] Gupta PC, Kota PB, Yerramsetty, et al. The supraclavicular approach to decompression of thoracic outlet. Semin Vasc Surg. 2024;37(1):57–65. 38704185 10.1053/j.semvascsurg.2024.01.006

[16] Panda N, Hurd J, Madsen J, et al. Efficacy and safety of supraclavicular thoracic outlet decompression. Ann Surg. 2023;278(3):417–25. 37334712 10.1097/SLA.0000000000005957

[17] Saglam M, Firat T, Vardar-Yagli N, et al. Respiratory dysfunction in individuals with thoracic outlet syndrome. J Manipulative Physiol Ther. 2020;43(6):606–11. 32829949 10.1016/j.jmpt.2019.10.006

[18] Annarumma G, Spinelli A, Serio A, et al. Forward head posture and neck disability: what is the effect on lung function? Explor Med. 2023;4:207–14.

[19] Chu E, Leung KY, Ng LLW, et al. Vascular thoracic outlet syndrome: a case report. J Contemp Chiropr. 2021;4:142–5.

[20] Trager RJ. Chiropractic and nontraditional treatment of NTOS, in thoracic outlet syndrome. Springer; 2021. pp. 229–40.

